# Effects of Heat Shock on Survival and Reproduction of Two Whitefly Species, *Trialeurodes vaporariorum* and *Bemisia tabaci* Biotype B

**DOI:** 10.1673/031.008.2401

**Published:** 2008-03-20

**Authors:** Xuhong Cui, Fanghao Wan, Ming Xie, Tongxian Liu

**Affiliations:** ^1^ State Key Laboratory for Biology of Plant Diseases and Insect Pests, Institute of Plant Protection, Chinese Academy of Agricultural Sciences, Beijing, China; ^2^ Center for Management of Invasive Alien Species, Ministry of Agriculture, Beijing, China; ^3^ Vegetable IPM Laboratory, Texas Agricultural Experiment Station, Texas A&M University System, Weslaco, Texas, 78596, USA

**Keywords:** sweetpotato whitefly, silverleaf whitefly, greenhouse whitefly, egg hatch, sex ratio

## Abstract

The effects of heat shock on survival and reproduction of two whitefly species, *Trialeurodes vaporariorum* (Westwood) and *Bemisia tabaci* (Gennadius) biotype B (Homoptera: Aleyrodidae), were compared in the laboratory. Whitefly adults were exposed to 26 (control), 37, 39, 41, 43 and 45°C for 1 hour, and were then maintained at 26°C. Adult survival was significantly affected when they were exposed at 41°C or higher for *B. tabaci* or 39°C or higher for *T. vaporariorum*. All males of *T. vaporariorum* were killed at 45°C. In both whitefly species, females were more tolerant to high temperatures at 39°C or higher than males. Female fecundity was not significantly different when *B. tabaci* adults were heat-shocked at all temperatures. In contrast, the fecundity of *T. vaporariorum* females declined with the increase of temperature, and only a few eggs were oviposited at 43°C. Survival or hatch rates of the F1 nymphs of both whitefly species declined as heat-shock temperature increased, and no *T. vaporariorum* nymphs were hatched at 43°C. Similarly, percentages of F1 offspring developing to adults for both whitefly species also declined as the heat-shock temperature increased. Sex ratios of the F1 offspring were not significantly affected for *T. vaporariorum* but were slightly affected for *B. tabaci* at 43 and 45°C. The significance of heat shock in relation to dispersal, distribution and population dynamics of the two whitefly species is discussed.

## Introduction

The temperature of insects is approximately the same as that of the environment, hence temperature has a profound effect on their colonization, distribution, abundance, behavior, life history and fitness (Cossins and Bowler 1987; Denlinger and Yocum 1998; James et al. 2002; Hoffmann et al. 2003). In addition, insects can be killed immediately after a short period of exposure to extremely high temperature, a method that has been widely used to control some economically important pests (Fields 1992; Mourier and Poulsen 2000). At a wide range of elevated temperatures below lethal high temperatures, insects can be affected by thermal injury, which may be manifested during later development stages, resulting in reduced survival and fitness (Denlinger and Yocum 1998). For example, in *Sarcophaga crassipalpis* and *Drosophila melanogaster*, the flies fail to emerge from the puparium if pupae or pharate adults suffered heat shock (Mitchell and Lipps 1978; Delinger et al. 1991). Developmental abnormalities and phenocopy mutations were observed in *D. melanogaster*, and some *Aedes* spp. experienced heat stress (Anderson and Horsfall 1963; Horsfall et al. 1964). Exposure to heat induced male sterility in *D. buzzatii*, *Ceratitis capitata*, and *Aedes* mosquitoes (Economopoulos 1996; Vollmer et al. 2004). *Ephestia cautella* and *Plodia interpunctella* experienced reduced fecundity after exposure to high temperature (Arbogast 1981). In transgenic *D. melanogaster*, the proportional hatch of eggs decreased if maternal females experienced heat shock, indicating the effects of heat shock continued into the next generation (Silbermann and Tatar 2000).

The potential of an insect population to flourish will depend in part on its size and the fitness of individuals. In nature, insects frequently experience fluctuating temperature regimes and these fluctuations may result in exposure to heat stress conditions that may, in turn, influence insect species distribution and population dynamics. *Trialeurodes vaporariorum* (Westwood) and *Bemisia tabaci* (Gennadius) biotype B (Homoptera: Aleyrodidae) are two of the most common and economically important whitefly species under field and protected conditions (Henneberry et al. 2002). *B. tabaci* is a tropical/subtropical species (Brown et al. 1995), and its recent outbreaks provide new evidence that the pest can protect itself from heat damage in extreme environments by residing under the leaf, utilizing heat shock proteins (Hsp70 and Hsp90) and raising levels of sorbitol in their blood by more than 8 fold under increased temperatures (Salvucci et al. 2000). This species is comprised of numerous biotypes, and each biotype differs in biological and virus transmitting characteristics (Brown et al. 1995; Perring 2001). Severe outbreaks usually subside eventually, but damage can be serious and must be managed by a combination of environmental manipulation, natural enemy enhancement and area-wide control programs.

*Bemisia tabaci* was found in China in 1949 and was not considered an important pest (Cho 1949) until the recent invasion of *B. tabaci* B biotype, which has been found in most provinces of China and become a severe pest of numerous field and ornamental crops (Luo et al. 2002). *T. vaporariorum* has been a major pest of vegetables and ornamental plants under protected environmental for many years, and it may coexist with *B. tabaci* on the same crop systems under protected conditions, but their population dynamics differ (Luo et al. 2004). Generally, the populations of *B. tabaci* reach their peak in summer whereas those of *T. vaporariorum* reach their peak under cooler climatic conditions (Luo et al. 2004). The temperature is normally above 30°C during summer months in northern China, and the highest temperature during the day could exceed 39°C (Climate Databases, Chinese Academy of Forestry, 1951–2000). The superior capacity of *B. tabaci* biotype B to survive at high temperatures favors population increases in hot weather, whereas the mild environmental conditions in the fall and winter could favor *T. vaporariorum*. To understand the effects of extreme temperatures on distribution, dispersal and population dynamics of these two whitefly species, we have conducted a series of studies to determine the effects of temperature, especially extreme temperatures on them. Here the effects of heat shock, a brief (1 h) exposure to high temperature, on the biological fitness of the two whitefly species is presented, including adult survival and reproduction, and survival and sex ratio of the F1 offspring.

## Materials and Methods

### Insects and host plants

*Bemisia tabaci* biotype B and *T. vaporariorum* used in this study were originally collected from tomato plants, *Lycopersicon lycopersicum* L. (Solanales: Solanaceae) in the experimental fields of the Chinese Academy of Agricultural Sciences, Beijing, from October to November in 2003. The *B. tabaci* colony was maintained on cotton plants, *Gossypium hirsutum* (L.) (Malvales: Malvaceae) (variety ‘Simian No. 3’), and the *T. vaporariorum* colony was maintained on green bean plants, *Phaseolus vulgaris* L. (Fabaceae: Faboideae) (cv. “Gong Geizhe”) in separate greenhouses under 20–34°C, 50–60% RH and natural photoperiod (39°55′ N, 116°20′ E) conditions. The plants were singly grown in plastic pots (9 cm in diameter) in greenhouses.

### Effects of heat shock on survival and reproduction of the two whitefly species

Newly emerged adults of *B. tabaci* and *T. vaporariorum* (≈3 h old) were collected and sexed under a stereomicroscope. A single adult was placed in a glass tube (13.3 mm in length × 10 mm in diameter). At least 50 female and male adults were assayed. The whiteflies inside the glass tubes were heat-shocked at each of the five temperature regimes, 37, 39, 41, 43 and 45 ± 0.2°C in climatic incubators (MHT350, Sanyo Electric Co., Ltd., Osaka, Japan) for 1 hour. The temperatures were selected based on normal temperatures in the greenhouses in northern China during summer. The 1-hour heat-shock duration was selected based on our preliminary experiments that this duration was sufficient to cause changes for the whiteflies. The adults maintained at 26°C were used as untreated controls. After the heat shock, the whiteflies were placed at 26°C for 2 hours to allow the adults to recover. The number of whiteflies recovered was checked. The adults were considered dead if all appendages did not move after touching with a brush. This experiment for each whitefly species was replicated 5 times.

Reproduction of the surviving adults for each whitefly species was further examined in climatic chambers at 26 ± 0.5°C, 60% RH and a photoperiod of 14:10: (L: D). Each pair of whiteflies (adult female and adult male) was confined on the lower leaf surface of a cotton plant for *B. tabaci* or green bean plant for *T. Vaporariorum* using a leaf clip-on cage (Zang et al. 2005). Live whiteflies inside the clip-on cages were transferred to another new leaf every 2 days. The leaves with whitefly eggs were cut from the plant after the whiteflies were transferred to a new leaf. The whitefly eggs were counted under a stereomicroscope. The process was continued until the female died. A new male was added if the previous one died during the experiment. Females that died within 24 h or produced no eggs were excluded from the analysis. The adults maintained at 26°C were used as untreated controls. At least 10 cages (replications) were used for each temperature regime for each whitefly species.

### Effects of heat shock on egg hatch and sex ratio of F1 progeny

After heat shock and recovery, five pairs of female and male adults were released into a cage that was attached to the underside of the leaf of the corresponding host plant for 5 days. The adults were then removed and the eggs were counted. The plants with eggs were placed in climatic chambers at 26 ±0.5°C, 60% RH and a photoperiod of 14:10 (L: D). The eggs were examined 4 d after oviposition, and number of hatched nymphs was counted. The eggs were continuously examined every 2 days until no nymphs hatched after four successive days. At the end of each census interval, the plants with whitefly eggs or nymphs were replaced in the climatic chambers until completion of adult eclosion. When the red-eyed nymph occurred on the leaf, the leaf was caged with a bag (10 × 10 cm) that was made of fine nylon mesh gauze (120 /cm^2^). After adult emergence, the bags with adults were collected immediately and sexed. Each treatment was replicated five times. The adults maintained at 26°C were used as untreated controls.

### Statistical analysis

Survival, preoviposition period, oviposition, nymph hatch, progeny that developed to adults and sex ratio were analyzed using analysis of variance (ANOVA), and means were separated using the Student-Newman-Keuls Test (SNK) at *P* < 0.05 (SAS Institute 2006).

## Results

### Effects of heat shock on survival of whitefly adults

**Figure 1.**
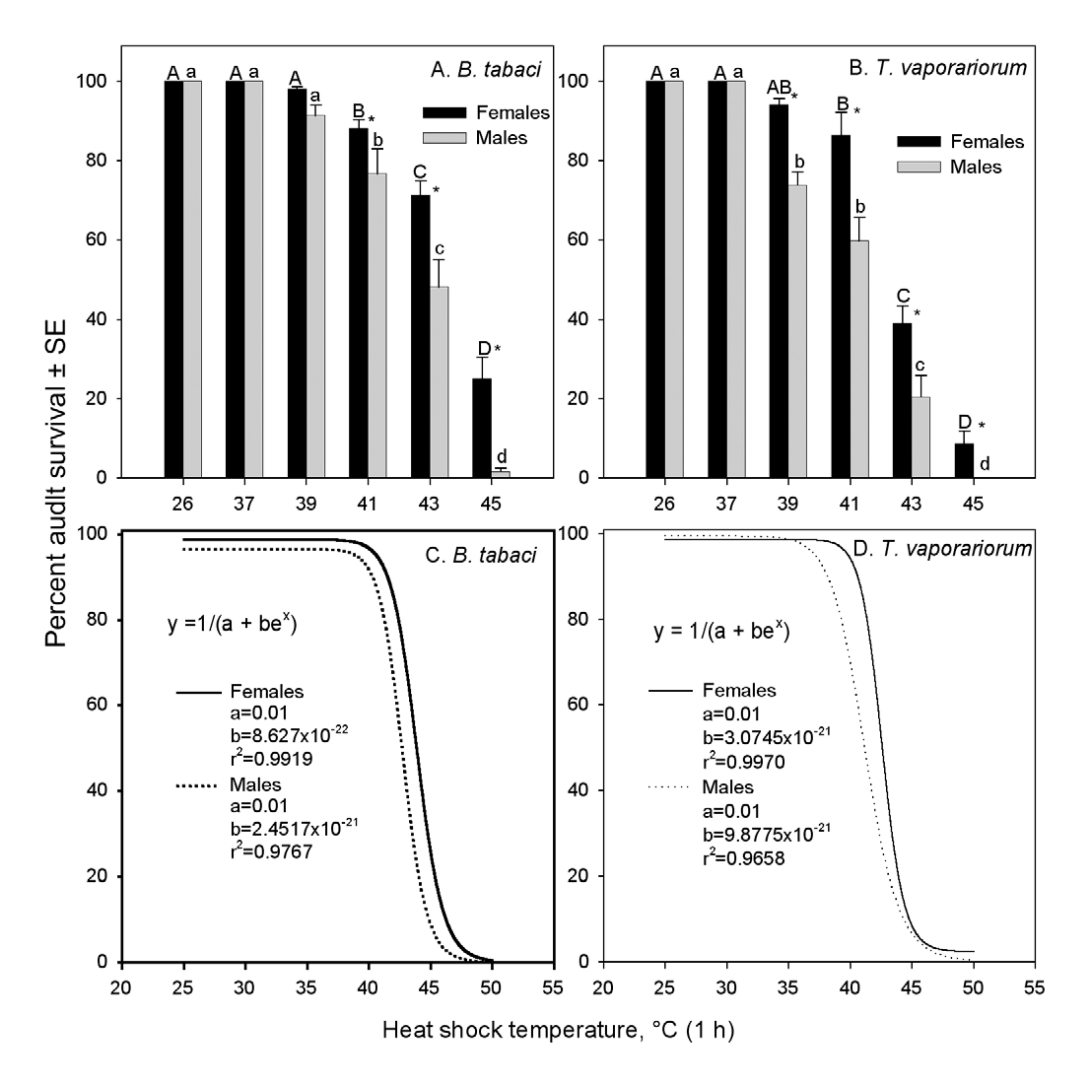
Relationship between survival rates of the female and male adults of *Bemisia tabaci* biotype B and *Trialeurodes vaporariorum* after exposure to different temperatures for 1 h. In A and B, the upper case letters over the bars indicate the survival rates at the six heat shock temperatures differ significantly at *P* <0.05 for females, and the lower case letter, for males for each whitefly species; the ‘*’ indicates that the survival rates differ significantly between females and males (SNK, SAS Institute 2006). In C and D, the curves show the relationship between heat-shock temperatures and survival of female and male adults of the two whitefly species, and fit an nonlinear model, y = 1/(a + be^x^); where, y is survival rate (%), and x is heat-shock temperature.

All adults of both *B. tabaci* and *T. vaporariorum* survived at 26°C (untreated control) and at 37°C (Figure 1), and those that were treated at 39°C and higher exhibited different responses in survivorship that varied with sex and whitefly species. The overall survival rates were significantly different between the whitefly species (*F* = 32.80; df = 1, 88; *P* < 0.0017), heat-shock temperatures (*F* = 370.77; df = 4, 88; *P* < 0.0001), interactions between species and heat-shock temperature (*F* = 7.29; df = 4, 88; *P* < 0.0001), gender (*F* = 71.52; df = 1, 88; *P* < 0.0001), interaction of heat-shock and gender (*F* = 4.71; df = 4, 88; *P* = 0.0017), and interaction of species x heat-shock temperature x gender (*F* = 2.79; df = 4, 88; *P* = 0.0313), but not the interaction between species and gender (*F* = 0.47; df = 1, 88; *P* = 0.4968). The survival rates of *B. tabaci* were different among the heat-shock temperatures (*F* = 173.68; df = 4, 42; *P* < 0.0001), gender (*F* = 30.74; df = 1, 42; *P* < 0.0001), and the interaction between heat-shock temperature and gender (*F* = 3.93; df = 4, 42; *P* = 0.0085). Similar results were found for *T. vaporariorum*, and there were significant differences among the heat-shock temperature (*F* = 203.64; df = 4, 46; *P* < 0.0001), gender (*F* = 40.85; df = 1, 46; *P* < 0.0001), and the interaction between heat-shock temperature and gender (*F* = 6.31; df = 4, 46; *P* = 0.0122).

The survival rates of both the female adults and the male adults of the two whitefly species were significantly different (*F* = 95.85–118.82; df = 5, 24–28; *P* < 0.0001), and exhibited similar responses to heat shocks. All whitefly adults survived at ambient temperature and the 37°C heat-shock treatment, but the survival rates declined as the heat-shock temperature increased. The relationship between the survival rates and heat-shock temperatures of females and males of both whitefly species fit well to a non-linear model of y = 1/(a+be^x^) (r^2^ ranged from 0.9659 to 0.9970; *P* < 0.0001) (Curve 2D 5.01, 2002; Systat Software Inc., San Jose, CA) (Figure 1).

**Figure 2.**
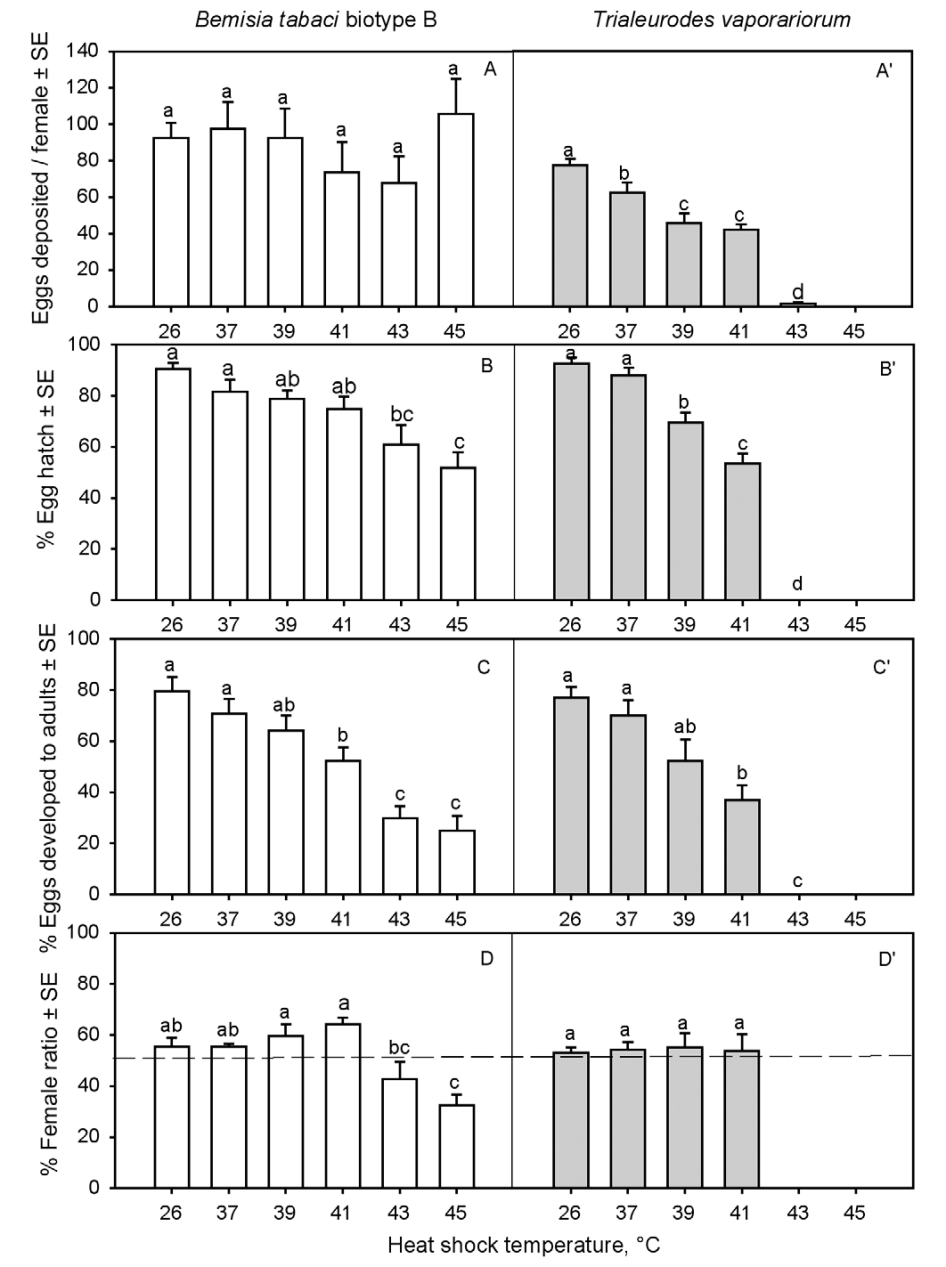
Effect of heat shock on major biological characteristics of *Bemisia tabaci* biotype B and *Trialeurodes vaporariorum*, including oviposition, nymph hatch rate, percentage of eggs that developed to adults, and female ratio. Different letters over the bars indicate the means at the six heat temperatures differ significantly for each biological parameter for each whitefly species at *P* < 0.05 (SNK, SAS Institute 2006).

For *B. tabaci*, there were no significant differences between the females and males at ambient temperature and following heat-shock treatments of 39 and 41°C (39°C: *F* = 4.79; df = 1, 10; *P* = 0.0535; 41°C: *F* = 2.87, df = 1, 8; *P* = 0.1288). However, when they were treated at 43 and 45°C, significantly less males survived after the heat shock than the females (71.3% vs 46.1% and 24.9% vs 1.696 for females and males at 43 and 45°C, respectively) (43°C: *F* = 10.54; df = 1, 8; *P =* 0.0017; 45°C: *F* = 16.61; df = 1, 8; *P* = 0.0036). For *T. vaporariorum*, the survival rates of males were significantly lower than the females following the heat-shock treatments of 39°C and higher (*F* = 5.85; df = 1, 8–12; *P* = 0.0419–0.0001).

### Effects of heat shock on preoviposition, reproduction, nymph hatch and sex ratio Preoviposition

Heat shock had no influence on preoviposition periods of the two whitefly species (*B. tabaci*: *F* = 1.17; df = 5, 238; *P* = 0.3262; *T. Vaporariorum*: *F* = 0.09; df = 5, 200; *P* = 0.9842), and the preoviposition period ranged from 2.0 to 2.2 d.

### Reproduction

Effects of heat shock on reproduction differed dramatically between the two whitefly species (*F* = 36.45; df = 1, 162; *P* < 0.0001), among the heat-shock temperatures (*F* = 2.84; df = 5,162; *P =* 0.0174) and the interaction between species and heat-shock temperature (*F* = 2.47; df = 5, 162; *P* = 0.0349) (Figures 2A and 2A'). Whereas heat shock did not exhibit any significant effects on numbers of eggs oviposited by the females of *B. tabaci* (*F* = 0.92; df = 5,104; *P* = 0.4698) (Figure 2A), it significantly reduced the number of eggs oviposited by the females of *T. vaporariorum* with the increase of temperature (*F* = 73.90; df = 5, 58; *P* < 0.0001) (Figure 2A'). The females of *T. vaporariorum* did not oviposit after heat shock at 45°C, and only a few at 43°C. The effects of the same heat shock temperature on the reproduction of the two whitefly species differed significantly. Number of eggs oviposited between the whitefly species did not differ significantly at 37 and 41°C (*F* = 2.08–3.08; df = 1, 26–28; *P* = 0.0911–0.1600), but fewer eggs were deposited by *T. vaporariorum* than by *B. tabaci* at 39, 43 and 45°C (*F* = 5.01–17.37; df = 1, 25–28; *P* = 0.0336–0.0001).

### Egg hatch

The proportion of nymphs hatched from the eggs of both *B. tabaci* and *T. vaporariorum* did not differ significantly between the two species (*F* = 0.06; df = 1, 48; *P* = 0.6921), but differed significantly among the heat-shock temperatures (*F* = 34.13; df = 5, 48; *P* < 0.0001), and the interaction between species and heat-shock temperature (*F* = 12.99; df = *5,* 48; *P* < 0.0001), with a tendency of declining egg hatch with increasing heat shock temperature (Figures 2B and B'). However, the effects of heat shock on hatching of the nymph from the egg differed between the two whitefly species. For *B. tabaci*, the percentages of eggs hatched did not decline significantly until the temperature increased to 43 and 45°C, whereas those for *T. vaporanorum* declined at 39°C, and no nymphs were hatched at 43 and 45°C.

### Survival to adults

The percentages of the progeny of both *B. tabaci* and *T. vaporanorum* that developed to adults were not significantly different between the two species (*F* = 0.56; df = 1, 48; *P* = 0.4582) nor the interaction between species and heat-shock temperature (*F* = 1.36; df = 5, 48; *P* = 0.2611), but differed significantly among the heat-shock temperatures (*F* = 24.75; df = 5, 48; *P* < 0.0001), with a tendency of declining the heat shock temperatures increased (Figure 2C and C'). The percentages of progeny that developed to adults of both *B. tabaci* and *T. vaporanorum* did not decline significantly until the temperature increased to 41°C and higher, and 29.8% and 25.1% of the progeny of *B. tabaci* developed to adults compared with no nymph survival for *T. vaporariorum* at 43 and 45°C, respectively.

### Sex ratio

Of the progeny of *B. tabaci* that developed to adults, different percentages of females were found among the different heat-shock treatments (*F* = 7.90; df = 5, 24; *P* = 0.0002) (Figure 2D). However, the female ratio was similar among the treatments at the ambient temperature (26°C) and the three heat shock treatments of 37, 39 and 41°C. Lower percentages of females were found at the two higher temperatures, 43 and 45°C. Of the progeny of *T. vaporanorum* that developed to adults, approximately equal proportions (53.0 to 55.2%) were females with no differences among the heat shock treatments (*F* = 0.04; df = 3, 23; *P* = 0.9881) (Figure 2D').

## Discussion

In ectothermic organisms, temperature plays a vital role in survival and reproductive success. When temperatures exceed an insect's optimum temperature range, there are two mutually exclusive results: survival or death (Denlinger and Yocum 1998). Even if a species could survive after exposure to heat stress, fitness is probably affected (Scott et al. 1997; Rinehart et al. 2000). Our results show that *B. tabaci* and *T. vaporariorum* adults exhibited different responses to heat-shock, although their post-heat shock survival at different temperatures fit a common model (Figure 1). The survival rates of female and male adults of *B. tabaci* biotype B were 100% at 26 and 37°C, and declined slowly until the temperature increased to 41°C. Above this temperature at 43 and 45°C, *B. tabaci* biotype B males had significantly lower survival rates than females. Similarly, *T. vaporariorum* survived 100% at 26 and 37°C, but the survival rates of *T. vaporariorum* declined more rapidly and sharply than *B. tabaci* biotype B at higher temperatures (>41°C), especially for males, whereas the survival rates for females were significantly higher at 39°C or higher.

Post-shock surviving whiteflies of both species also exhibited a different response in fecundity. After heat shock, the number of eggs deposited by *B. tabaci* was not significantly different at the six temperatures (Figure 2A), whereas the number of eggs deposited by *T. vaporanorum* decreased at 37°C and above (Figure 2A'). These results indicate that *B. tabaci* was able to tolerate higher temperatures than *T. vaporariorum. T. vaporariorum* almost ceased depositing eggs after heat shock at 43°C. It has been reported that heat shock can cause injury to oocytes and ovarian development in females that could lead to the decrease in egg production. Heat shock can reduce male fertility due to direct injury to the testes and sperm (Chihrane and Lauge 1994, 1997; Krebs and Loeschcke 1994; Scott et al. 1997; Rinehart et al. 2000).

Heat-shock also affected the survival of the deposited eggs in the F1 generation as shown by the lower egg hatch (Figures 2B and 2B'). Although heat-shock did not affect oviposition of *B. tabaci*, it resulted in reduced egg hatch when temperatures were at 43°C or higher (Figure 2B). Heat shock severely reduced hatch of *T. vaporanorum* eggs with the increase in temperature, and no eggs hatched at 43°C (Figure 2B'). Similarly, the numbers of adults that eclosed from the F1 offspring were significantly reduced for both whitefly species (Figures 2C and C'). Less than 30% of *B. tabaci* offspring developed to adults at heat shock temperatures of 43 and 45°C (Figure 2C). Similar results have been reported in the literature. Geng et al. (2005) found that when the middle and late pupal stages of *Trichogramma dendrolimi* Matsumura heat-shocked for 6 hours at 35 and 40°C, emergence rate and number of wasps per host egg were significantly reduced and nearly all pupae failed to develop to adults. However, they also found that heat shock of the pupal stage had little effect on progeny.

The effects of heat shock on sex ratios of the two whitefly species were also different (Figure 2D and D'). Heat shock at 41°C did not alter the sex ratio of the F1 progeny of *B. tabaci*, but fewer females emerged when the parent females were heat-shocked at 43 and 45°C (Figure 2D). In contrast, sex ratios of the F1 progeny of *T. vaporariorum* were not affected after the parent females were heat-shocked (Figure 2D'). It has been reported that changes in sex ratios may affect the population dynamics of the insect (Krainacker and Carey 1988; Horiwitz and Gerling 1992). In our study, both males and females that had been subjected to heat shock at 43 and 45°C were paired together. As a result, we could not determine if both sexes were affected, or if perhaps the male or female alone were affected. As shown in Figure 2A, the number of eggs laid per female that were heat-shocked at 43 and 45°C was not reduced, but the ratio of female progeny was significantly reduced (Figure 2D), suggesting that male fertility may have been more affected by the high temperatures because *B. tabaci* is haplodiploid.

There is no doubt that food supplies play vital roles for the two species of whiteflies. Salvucci (2000) found that with a source of food supply (cotton leaves), sorbitol levels, that protect proteins from heat stress, increased in *B. tabaci* by 20 fold at 40°C compared with 25°C. However, an acclimation period is required for the whiteflies to adjust to the new conditions, for example, by raising sorbitol levels after heat shock. In the present study, we found that it took an average of 12.3±0.03, 12.3±0.1, 12.4±0.07, 15.1± 0.07, and 18.4 ± 0.08 min in the climatic incubators to reach 37, 39, 41, 43, and 45°C, respectively, from 26°C. It is possible that the whiteflies were able to raise their sorbitol level in response to the increasing temperature.

Heat shock proteins (Hsp) play an important role in tolerating heat stress in insects (Solomon et al. 1991; Feder et al. 1996) and the difference in tolerance may be related to differences in their optimal temperature for the heat shock protein-induction response (Lindquist 1986; Goto and Masahito 1998). For most organisms, heat shock proteins are synthesized when ambient temperatures exceed the normal temperature optimum of the organism (Parsell and Lindquist 1993). However, studies have shown that whiteflies accumulate polyhydric alcohols (polyols), such as sorbitol, in response to high temperature (Wolfe et al. 1998; Hendrix and Salvucci 1998; Salvucci et al. 1999, 2000). For whiteflies, accumulation of sorbitol occurs under field conditions in response to a diurnal increase in temperature (Wolfe et al. 1998). Sorbitol and other polyols are well-known solvent modifiers that protect the native structure of proteins from thermal denaturation (Back et al. 1979; Henle et al. 1982). Recent evidence with whiteflies is consistent with the idea that sorbitol acts as a thermoprotectant *in vivo*, protecting whitefly proteins from thermal denaturation (Salvucci 2000). Salvucci et al. (2000) found that Hsp70 and Hsp90 were the major polypeptides synthesized by whiteflies in response to heat stress. The amounts of Hsp70 and Hsp90 protein and Hsp70 mRNA were unchanged in response to a diurnal increase in temperature, whereas sorbitol content increased 8-fold. That steady-state levels of heat shock proteins did not increase with higher rates of synthesis suggests that they turn over faster in heat-stressed whiteflies. Hsp70 transcript levels were highest when nutrient deprivation accompanied heat stress. Thus, heat shock proteins appear to be especially important for heat-stressed whiteflies when sorbitol synthesis is limited nutritionally.

Examination of the recent outbreaks of *B. tabaci* employing new molecular tools resulted in taxonomic findings that showed this pest contained numerous biotypes (Gawel and Barlett 1993; Perring et al. 1993; Guirao et al. 1997; De Barro et al.1997,^1998^,^2000^). The appearance of these biotypes was probably related to the very large populations that developed, their vast ranges and host plant varieties, to various selective pressures and to the physiological characteristics of the species. In addition, these responses coincide with their adaptation to dispersal range, spatial and temporal distributions, and different environments.

In nature, population dynamics varies in different temporal and spatial niches, reflecting different requirements for thermal budget and habitat traits (Stanley et al. 1980; Stratman and Markow 1998; Chen and Kang 2002). Therefore, the ability of insects to survive thermal stress, together with other factors, plays an important role in determining distribution of a species (Bale et al. 2002). Tsueda and Tsuchida (1998) reported that *B. tabaci* had a higher development rate, survival rate and oviposition than that of *T. vaporariorum* at 30°C, indicating that *B. tabaci* has higher viability than *T. vaporanorum* at less severe high temperatures. Our results provide further information explaining the mechanisms and phenomena of population dynamics of the two whitefly species in northern China as reported by Luo et al. (2004). The results would be very useful for monitoring population dynamics, distribution and dispersal of the two whitefly species, and for developing sustainable and integrated strategies for the management of the two important pests.
